# The association between anti-Müllerian hormone and vitamin 25(OH)D serum levels and polycystic ovarian syndrome in adolescent females

**DOI:** 10.1186/s12958-020-00676-y

**Published:** 2020-11-21

**Authors:** Samantha Simpson, David B. Seifer, Veronika Shabanova, Anna Y. Lynn, Catherine Howe, Erin Rowe, Sonia Caprio, Alla Vash-Margita

**Affiliations:** 1 Department of Obstetrics, Gynecology, and Reproductive Sciences, Yale School of Medicine, 310 Cedar Street, New Haven, CT 06520-8063 USA; 2 Department of Biostatistics, Yale School of Public Health, New Haven, CT USA; 3 Yale School of Medicine, New Haven, CT USA; 4 Department of Radiology and Biomedical Imaging, Yale School of Medicine, New Haven, CT USA; 5 Department of Pediatric Endocrinology and Diabetes, Yale School of Medicine, New Haven, CT USA

**Keywords:** Polycystic ovarian syndrome, Adolescents, Anti-Müllerian hormone, Vitamin D

## Abstract

**Background:**

High anti-Müllerian hormone (AMH) levels and 25-hydroxyvitamin D [25(OH)D] deficiency have been associated with polycystic ovarian syndrome (PCOS) in adult women, and implicated in its pathogenesis. Herein we determined if the level of both AMH and 25(OH)D are altered in adolescent females with clinical features of PCOS.

**Methods:**

This is a cross-sectional study utilizing a retrospective chart review of 128 patients aged 12–20 referred to an academic adolescent gynecology and endocrinology clinic for an evaluation of suspected PCOS. Unadjusted comparisons of AMH and 25(OH)D distributions between subjects with and without PCOS were performed using the Wilcoxon Rank Sum test. Quantile regression was used to compare the median AMH and 25(OH)D between subject groups; adjusting for race, ethnicity, BMI, insurance type, age, and season when bloodwork was performed.

**Results:**

Seventy-four subjects were classified as having PCOS by meeting ≥2 of the three Rotterdam diagnostic criteria, and 47 subjects met only one Rotterdam diagnostic criteria, and were used as the comparative non-PCOS group. There were statistically significant unadjusted differences in median levels of AMH and 25(OH)D. In the adjusted analyses, median AMH was significantly higher in the PCOS group compared to the non-PCOS group (+ 2.39 ng/mL, 95% CI 0.43, 4.35, *p* = 0.018); 25(OH)D was significantly lower in the PCOS group (− 9.01 ng/mL, 95% CI -14.49, − 3.53 *p* = 0.001). In our sample, adolescents in both groups had insufficient 25(OH)D level (22 ng/mL) and elevated BMI (32.2 kg/m2).

**Conclusions:**

Adolescents with PCOS display high levels of AMH and low 25(OH)D levels. Since traditional clinical markers of PCOS may be physiologic in adolescents, AMH and 25(OH)D may be used as surrogate markers of PCOS risk in adolescents.

## Background

Diagnosis of polycystic ovarian syndrome (PCOS) in the adolescent population remains imprecise. In adults, the Rotterdam criteria – a diagnosis of exclusion in which two of three symptoms must be present, including oligomenorrhea or amenorrhea, clinical or biochemical evidence of hyperandrogenism, and polycystic appearing ovaries - are a commonly used algorithm to determine if a patient meets criteria for the PCOS diagnosis and warrants additional work up and ongoing surveillance for the syndrome’s sequelae [1, 2]. Adolescents may incur the health risks of PCOS, but the adult diagnostic criteria may be difficult to apply to this younger population [3–6]. Adolescents are more likely to have a physiologically long intermenstrual interval which can persist for up to 5 years after menarche due to immaturity of the hypothalamic-pituitary axis [6–8]. Adolescents are more likely to have acne, with up to 29.5% of pubertal girls exhibiting significant inflammatory acne without concomitant differences in testosterone when compared to their acne-less peers [9]. Additionally, there is disagreement about the definition of polycystic-appearing ovaries in the adolescent population. The Rotterdam criteria use ovarian volume greater than 10 mL or more than 12 follicles on an ovary using a vaginal transducer, however, adolescents have been noted to have greater ovarian volumes and follicular counts than adult women, and it is not recommended to perform vaginal ultrasound on this population [6, 10, 11]. In a retrospective study, only 38% of adolescents who met Rotterdam criteria for PCOS had ovarian volumes ≥10 mL, while 7% of a comparison non-PCOS population had ovarian volumes ≥10mL [12]. In a prospective study, 35% of an unselected adolescent population had polycystic ovarian appearance [6]. Recent guidelines recommend against using ultrasound at all in adolescents for diagnosis of PCOS until 8 years post-menarche [5]. However, similar to adult women, we know that larger ovaries and higher antral follicle counts in adolescents are associated with elevated androgens [13].

In adult women, it has been noted that women with PCOS tend to have a higher AMH than those without PCOS [14–16]. This is biologically plausible as AMH is secreted by the granulosa cells of pre-antral and small antral ovarian follicles [17]. It has been suggested that AMH may be a better determinant of the overabundance of ovarian follicles typical of PCOS than antral follicle count, as ultrasound is dependent on the quality of the ultrasound and the interpreter [18]. Higher AMH levels have been associated with greater menstrual disturbances and greater ovarian volume [15, 19, 20]. Serum AMH cutoffs for PCOS in adults have been proposed, ranging from 3.94 ng/mL with area under the curve (AUC) of 0.916 and 89.8% specificity and 80% sensitivity [21], 4.45 ng/mL with AUC of 0.870 and 74.6% specificity and 76.1% sensitivity [22], to 5.72 ng/mL with AUC of 0.77 [19]. A meta-analysis settled on an AMH cutoff of 4.7 ng/mL with AUC 0.87 with specificity of 79.4% and sensitivity of 82.8% [23]. In adolescents, the average AMH tends to be higher. Previous studies in adolescents identified a cutoff of 7 ng/mL with AUC of 0.87 [24], and a cutoff of 6.26 with AUC 0.788 [25]. These studies have been criticized, however, for comparing obese oligomenorrheic girls with thin, normally menstruating counterparts.

Deficiency of 25-hydroxyvitamin D [25(OH)D] has been implicated as a mitigable factor in PCOS symptomatology [26–28]. Supplementation of 25(OH)D has been shown to decrease serum triglycerides, serum androgen levels, liver markers, hirsutism, insulin resistance, and normalize menstrual cycles in adult women with PCOS [26, 29–34]. 25(OH)D insufficiency (20-29 ng/mL) and deficiency (less than 20 ng/mL) is defined the same in adolescents and adults [35]. In adolescents, the association between 25(OH)D deficiency and PCOS is not as straightforward, with some small studies failing to find any connection [36, 37].

Molecularly, AMH and 25(OH)D are related [38–40]. AMH expression is regulated by 25(OH)D binding to the vitamin D receptor site in animals and humans, though the nature of this regulation has not been clearly elucidated [41, 42]. Studies in human prostate cell lines showed that the promoter region for the AMH gene contains a 25(OH)D response element [43]. AMH and 25(OH)D have been studied together clinically in adult women with and without PCOS and no consistent relationship has been found, but these two serum analytes have not been evaluated together in the adolescent population [38, 44, 45]. If we could establish a significant clinical difference in these hormone concentrations between adolescents with PCOS and a comparative group, this would strengthen AMH’s ability to be used as a biomarker for PCOS in the diagnostically challenging adolescent population. It would also identify a mitigable analyte, 25(OH)D, which could potentially open new treatment pathways for adolescents with PCOS. In this retrospective cross-sectional study, we aimed to determine if adolescents who met Rotterdam criteria for PCOS had different median levels of AMH and 25(OH)D, when compared to adolescents who met one aspect of the Rotterdam criteria but did not ultimately meet the current diagnostic standard for PCOS.

## Methods

### Study population

This retrospective study was approved by our institutional review board (Yale University IRB # 2000024068). We conducted a chart review of serial consecutive patients who were referred to the adolescent gynecology and endocrinology clinic (AGEC) for concern for PCOS (presenting complaints of oligomenorrhea, amenorrhea, hirsutism, acne, or elevated androgens) from November 2017 to December 2019. Inclusion criteria were a referral to AGEC, age range 12–20 years, and being at least 24 months post-menarche [46]. Patients were excluded if their oligomenorrhea was due to continuous hormonal contraceptives (such as medroxyprogesterone acetate intramuscular injections or continuous combined hormonal pills), if they had overt thyroid dysfunction or abnormal prolactin values, or had other causes of hyperandrogenism such as congenital adrenal hyperplasia or adrenal or ovarian tumor (evaluated via 17-hydroxyprogesterone value and the pelvic ultrasound in patients with elevated testosterone, or adrenal CT in patients with elevated serum DHEA-S).

### Data collection

For each eligible patient, we abstracted data from their clinical evaluation, which included a complete medical history, physical exam (though not necessarily a gynecologic exam), bloodwork according to the patient’s presenting symptom, and transabdominal pelvic ultrasound at the discretion of the attending physician. All subjects included in this retrospective chart review had relevant laboratory work and their transabdominal ultrasound performed within 6 months of their initial visit, before treatment for PCOS was initiated. Demographic variables extracted from the medical records were age, race, ethnicity, and insurance type. Clinical and medical variables were body mass index (BMI), age at menarche, presence of acne or hirsutism, levels of hormones, season of 25(OH)D bloodwork, and transabdominal ultrasound measurements of right and left ovaries.

### Laboratory assays

Due to the retrospective nature of this review, there was some heterogeneity in methods of laboratory assay. Quantification of 25(OH)D (one of two primary outcomes) was performed by liquid chromatography-tandem mass spectrometry (reference range 5.2-311 ng/mL, intra-assay coefficients of variance (CoV) 3.9–6.9%, inter-assay CoV 7.2%) [47] and by immunoassay (reference range 4–150 ng/mL, intra-assay CoV 2.3–5.4%, inter-assay CoV 7.8–10.6%) (DiaSorin; Saluggia, Italy); for both, laboratory standards considered values < 30 ng/dL evidence of 25(OH)D deficiency. AMH (one of two primary outcomes) was quantified by chemiluminescent immunoassay (reference range 0.08–24 ng/mL, intra-assay CoV 1.8–2.3%, inter-assay CoV 0.9–4.4%) (Beckman-Coulter; Brea, CA). Androstenedione was quantified by chromatography / mass spectrometry. DHEA and DHEA-S were quantified by immunoassay. Total testosterone was measured by competitive chemiluminescent immunoassay, and free testosterone was calculated based on measured total testosterone and measured sex hormone binding globulin. AMH is routinely ordered on adolescent females presenting for PCOS evaluation at our clinic, and 25(OH)D is routinely ordered in all overweight adolescents, as our clinic is in the Northern United States and there is a high prevalence of vitamin D deficiency and insufficiency.

### Ultrasound interpretation

All available ultrasound images done as part of the patient’s workup were re-interpreted by a single blinded university-affiliated pediatric radiologist who re-measured ovarian volume (0.5 x length x width x thickness) [48] and assessed if follicles (2-9 mm) were peripherally distributed or not. Due to lack of real-time imaging, the radiologist was unable to assess total number of follicles. Ovaries were considered polycystic appearing if follicles were peripherally distributed and one or both ovaries had a volume ≥ 10 mL.

### Comparator group assignment

Patients were classified as having PCOS if they met two or three of the Rotterdam criteria (oligomenorrhea or amenorrhea, hyperandrogenism, or polycystic-appearing ovaries) [1] and those who met only one of the Rotterdam criteria were considered as a comparator group, called non-PCOS in this manuscript. This clinical approach has been previously described by other PCOS researchers [49]. The oligomenorrhea / amenorrhea criterion was met if the intermenstrual interval was > 35 days for a year, or if the subject did not menstruate > 6 months if she previously had menstruated. Hyperandrogenism criterion was fulfilled if the patient had moderate to severe hirsutism, moderate to severe inflammatory facial or body acne, or total testosterone > 40 ng/dL, free testosterone > 3.6 pg/mL, or dehydroepiandrosterone-sulfate (DHEA-S) > 430 μg/dL. Polycystic appearing ovaries determined by the independent blinded radiologist as described above.

### Analysis

For the unadjusted analysis, demographic information, clinical findings, and laboratory values between group comparisons were made with Chi-square for categorical variables and Wilcoxon Rank-Sum tests for continuous and scale variables.

Adjusted analysis of the median levels in each primary outcome of interest (AMH and 25(OH)D) was performed using quantile regression, with PCOS diagnosis as the primary predictor, adjusting for race and ethnicity, private insurance status, BMI, age at evaluation, and season of 25(OH)D bloodwork. The adjustments were done with clinically meaningful variables, no matter their statistical significance, but we noted associations at alpha of 0.15. Results from the adjusted analysis were summarized using the effect size, the between-group difference in median levels of primary outcomes, with 95% confidence intervals (CI) obtained using the percentile method (2.5th and 97.5th percentiles) from 500 bootstrapped samples. The null hypothesis of no association between group (PCOS vs. non-PCOS) and each primary outcome was tested at the two-sided alpha of 0.05. No adjustment for multiple comparisons was done, as these were two different hypotheses. Analyses were formed using SAS 9.4 (Cary, NC).

## Results

### Study population

**Fig. 1:**
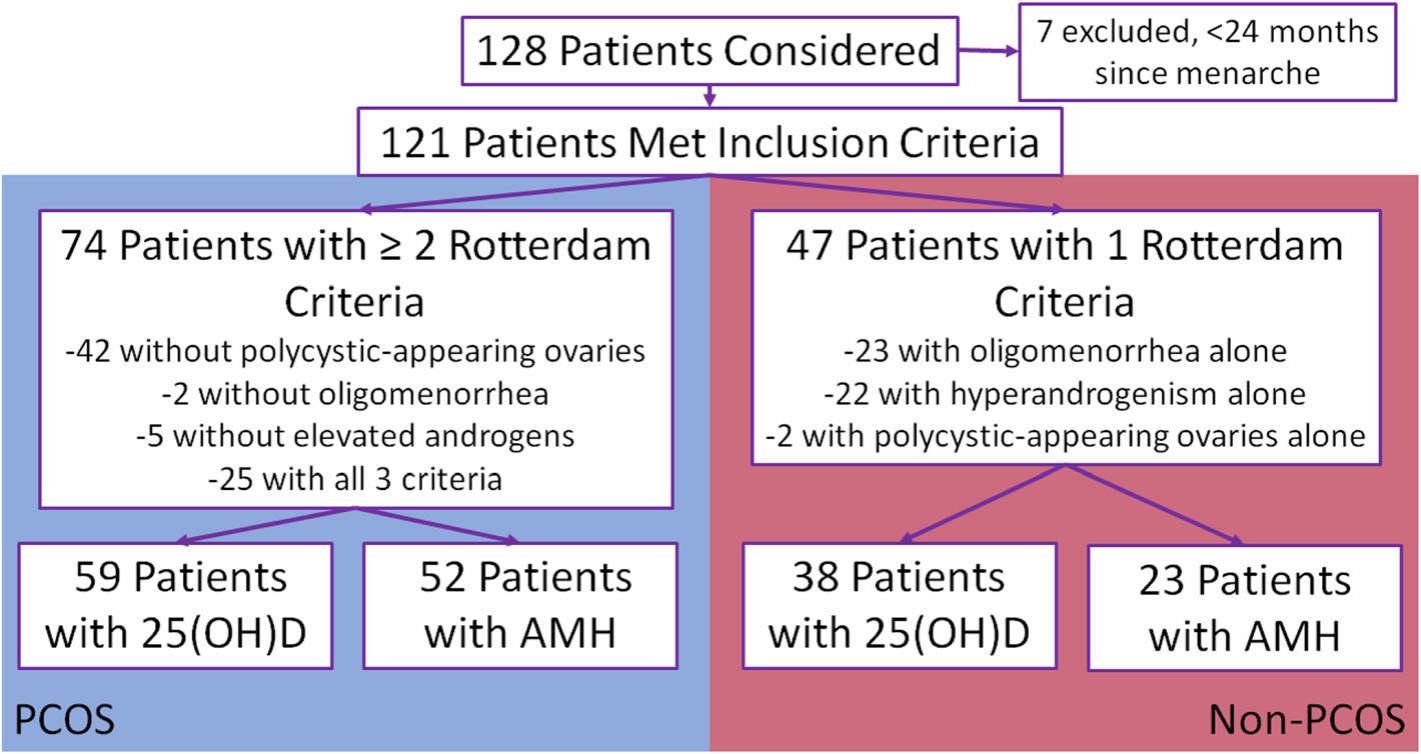
Consort diagram of patients included in analyses

Out of 128 patients who were referred to AGEC with concern for PCOS, 7 patients presented within 2 years of menarche and were excluded [46]. After evaluation, 74 patients (61.2%) were found to meet two or three of the three Rotterdam criteria (PCOS group), and 47 (38.8%) were found to only meet one criterion (non-PCOS group) (Fig. 1). Of these patients, 59 (79.7%) in the PCOS group and 38 (80.9%) in the non-PCOS group had 25(OH)D testing; 52 (71.3%) in the PCOS and 23 (48.9%) in the non-PCOS group had AMH testing (Fig. 1). Patient demographic information was compared between the PCOS and non-PCOS groups as a whole, and between patients who had AMH and 25(OH)D results and those who did not, to check for systematic selection bias due to missing data in the primary outcomes (Table 1). In the demographic factors, the only important difference was in the PCOS versus non-PCOS group in the percent of patients reporting oligomenorrhea, which was expected, as this alone is one of the diagnostic criteria for PCOS.

**Table 1:** Demographic information by PCOS status, AMH status, and 25(OH)D status

	PCOS (*n* = 74)	Non-PCOS (*n* = 47)	*P*	AMH (*n* = 75)	No AMH (*n* = 46)	*P*	25(OH)D (*n* = 97)	No 25(OH)D (*n* = 24)	*P*
Age at Evaluation	16.1 ± 1.8	15.7 ± 1.4	0.17	15.9 ± 1.6	16.0 ± 1.7	0.68	15.9 ± 1.7	16 ± 1.6	0.74
Age at Menarche	11.5 ± 1.8	11.1 ± 1.5	0.21	11.3 ± 1.6	11.5 ± 1.8	0.55	11.3 ± 1.7	11.5 ± 1.6	0.76
BMI	31.9 ± 8.1	32.6 ± 11.1	0.72	31.3 ± 9.6	33.6 ± 8.9	0.18	32.6 ± 9.4	30.2 ± 9	0.26
Race									
Caucasian	31 (42%)	20 (43%)	0.73	29 (39%)	22 (48%)	0.64	40 (41%)	11 (46%)	0.67
Black	11 (15%)	9 (20%)		10 (13%)	10 (22%)		14 (15%)	6 (25%)	
Other/No Answer	32 (43%)	18 (37%)		36 (48%)	14 (30%)		43 (44%)	7 (29%)	
Ethnicity									
Latina	19 (26%)	15 (32%)	0.61	18 (24%)	16 (35%)	0.43	28 (29%)	6 (25%)	0.49
Not Latina	44 (59%)	28 (60%)		44 (59%)	28 (61%)		55 (57%)	17 (71%)	
No Answer	11 (15%)	4 (8%)		13 (17%)	2 (4%)		14 (14%)	1 (4%)	
**Oligomenorrhea/ Amenorrhea**	**72 (97%)**	**23 (49%)**	**< 0.001**	61 (81%)	34 (74%)	0.45	76 (78%)	19 (79%)	0.99
Acne	32 (43%)	12 (26%)	0.17	30 (40%)	14 (30%)	0.9	35 (36%)	9 (38%)	0.8
Hirsutism	37 (50%)	15 (32%)	0.1	34 (45%)	18 (39%)	0.71	42 (43%)	10 (42%)	0.79
Private Insurance	31 (42%)	19 (40%)	0.97	32 (43%)	18 (39%)	0.59	38 (39%)	12 (50%)	0.44

Values given as Mean ± Standard Deviation, or Number (Percent). Age in years, BMI in kg/m^2^

### Unadjusted analysis

**Fig. 2:**
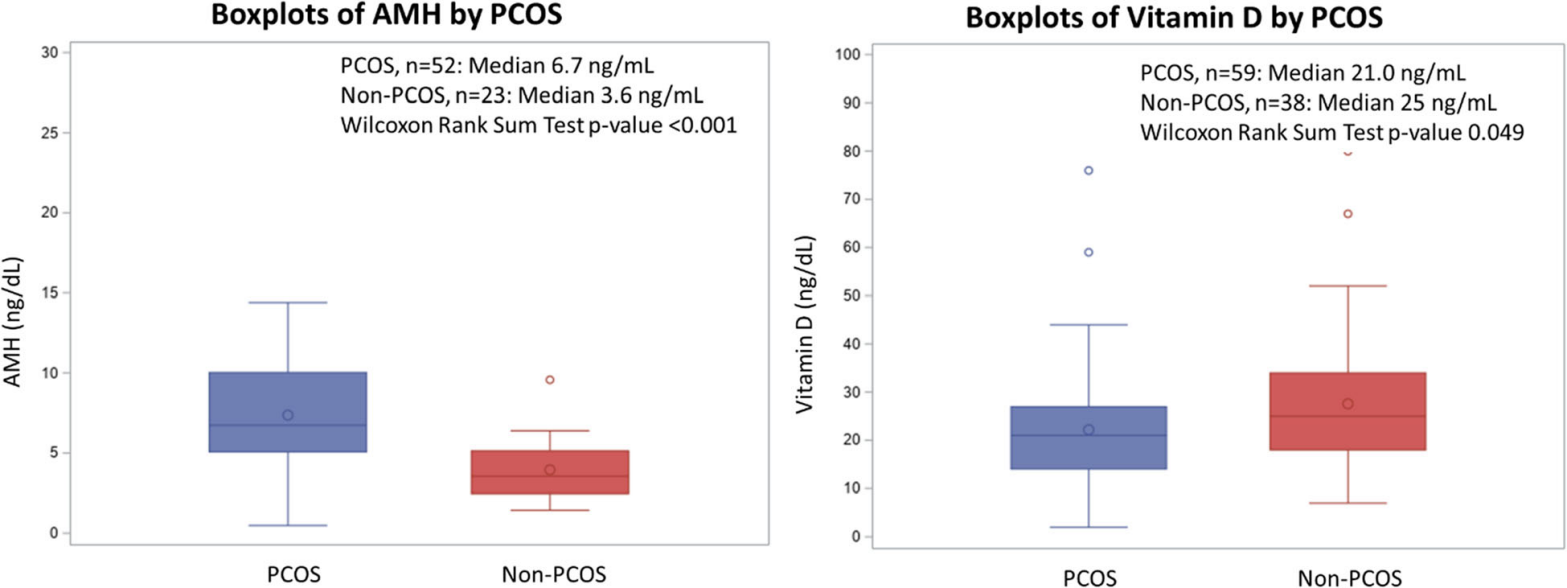
Boxplots of AMH and 25(OH)D based on PCOS status

Compared to adolescents in the non-PCOS group, median AMH was higher in the PCOS group (6.7 ng/mL v 3.6 ng/mL, *p* < 0.001), while median 25(OH)D was lower in the PCOS group (21 ng/mL v 25 ng/mL, *p* = 0.049) (Fig. 2). Of the 121 patients in the study, 91 (75.2%) completed ultrasound evaluation with images that were available for review by the independent blinded radiologist. There were no differences in mean right and left ovarian volumes between the PCOS and non-PCOS groups, but more patients in the PCOS group had peripherally distributed follicles (Table 2).

**Table 2:** Unadjusted analysis

	PCOS	n	Not PCOS	n	*P*
**AMH – ng/dL**	**6.7 (0.5–14.4)**	**52**	**3.6 (1.4–9.6)**	**23**	**< 0.001**
**25(OH)D – ng/mL**	**21 (2–76)**	**59**	**25 (7–80)**	**38**	**0.049**
**Testosterone, Total – ng/dL**	**47.5 (5.2–133)**	**70**	**33 (8.0–118.0)**	**42**	**< 0.001**
**Testosterone, Free – pg/dL**	**7.2 (1.6–24.4)**	**68**	**4.7 (1.4–24.3)**	**41**	**< 0.001**
DHEA-S - μg/dL	243 (46–869)	64	235 (86–697)	36	0.38
**17-Hydroxyprogesterone – ng/dL**	**57 (8–261)**	**51**	**45 (4–187)**	**29**	**0.05**
Ovarian Volume – Right, cc	9.2 ± 4.4	57	10.3 ± 15.1	34	0.69
Ovarian Volume – Left, cc	16.2 ± 51.1	56	12.5 ± 28.1	34	0.66
**Peripheral Follicle Distribution?**	**17 (23%)**		**4 (8.5%)**		**0.045**
Luteinizing Hormone – IU/L	11.5 (1.8–46.6)	50	9.5 (1.6–28.1)	17	0.34
Follicle Stimulating Hormone – IU/L	5.7 (2.5–11.1)	51	5.2 (1.3–7.9)	17	0.3

Clinical parameters given as Mean ± Standard Deviation or Number (Percent). Laboratory Values given as Median (Range)

Total and free testosterone was available in 112 (92.6%) patients. Patients with PCOS had higher median total (47.5 ng/dL v 33 ng/dL, *p* < 0.001) and free (7.2 pg/mL v 4.7 pg/mL, p < 0.001) testosterone, and higher median 17-hydroxyprogesterone (57 ng/dL v 45 ng/dL, *p* = 0.05). Androstenedione, luteinizing hormone, follicle stimulating hormone, and sex hormone binding globulin were comparable between the two groups of patients (Table 2).

### Adjusted analysis

Differences in median levels of both AMH and 25(OH)D persisted in the adjusted models (Table 3). Adolescents with PCOS had significantly lower median levels of 25(OH)D (− 9.01, 95%CI -14.49, − 3.53), but significantly higher median levels of AMH (2.39, 95%CI 0.43, 4.35). Of interest, a few statistical trends were noted: Latina subjects had lower median levels of both AMH and 25(OH)D; higher BMI was associated with lower median AMH; and median levels of 25(OH)D tended to be higher during the summer.

**Table 3:** Adjusted analysis with quantile regression

	Adjusted Difference in Median of AMH	*P*	Adjusted Difference in Median of 25(OH)D	*P*
PCOS	**2.39 (95% CI 0.43, 4.35)**	**0.018**	**−9.01 (95% CI − 14.49, −3.53)**	**0.001**
Age at Evaluation	−0.37 (95% CI −1.01, 0.26)	0.24	1.82 (95% CI −0.49, 4.13)	0.12
BMI	−0.11 (95% CI − 0.22, 0.003)	0.06	0.02 (95% CI − 0.31, 0.35)	0.9
Race (Black Reference)				
Caucasian	−0.89 (95% CI −4.50, 2.73)	0.62	5.02 (95% CI −7.14, 17.17)	0.41
Other/No Answer	3.59 (95% CI −0.18, 7.36)	0.06	2.61 (95% CI −9.82, 15.04)	0.68
Ethnicity (Latina Reference)				
Not Latina	**3.83 (95% CI 0.76, 6.90)**	**0.016**	9.56 (95% CI −0.37, 19.49)	0.059
No Private Insurance	0.81 (95% CI −1.58, 3.21)	0.5	−0.25 (95% CI −7.40, 6.89)	0.94
25(OH)D Season (Summer Ref)	–	–		
Winter			−14.20 (95% CI −30.47, 2.07)	0.086
Fall			−13.02 (95% CI −29.14, 3.10)	0.112
Spring			−9.92 (95% CI − 27.47, 7.63)	0.09

Values given as Median Difference (95% CI)

## Discussion and conclusions

To our knowledge, this is the first retrospective study to evaluate levels of AMH and vitamin 25(OH)D in an adolescent cohort concomitantly. We have identified that AMH is significantly higher in adolescent females with PCOS (6.7 ng/mL v 3.6 ng/mL), and our values are consistent with previously published reports that suggest AMH “cutoffs” around 6.26–7 ng/mL [24, 25]. The prior studies used thin, normally menstruating adolescents as a comparator group to the PCOS population and there was concern that AMH would not be able to be used to actually discriminate the diagnosis of PCOS in a “real world” application. In our study, we used a robust comparator population – these were not healthy adolescents without complaints related to their reproductive health; these were girls with one clinical or biochemical marker used in the diagnosis of PCOS. As the most recent guidelines recommend against the use of the ultrasound criterion in adolescents until 8 years post-menarche [5], adolescents who are at risk for PCOS may benefit from evaluation of AMH as a non-invasive marker for PCOS. This has biologic plausibility as AMH serves as a surrogate marker of ovarian follicle overabundance, and providers would not rely on the skill of the ultrasonographer performing a transabdominal ultrasound in an increasingly obese adolescent PCOS population [18].

We have also identified lower 25(OH)D concentration in adolescent girls with PCOS. This association was confirmed in the adjusted analysis when taking into account the season during which serum 25(OH)D was collected, as well as age, BMI, ethnicity, race, and insurance status. By demonstrating the clinically meaningful and statistically different lower serum 25(OH)D (by 9 ng/mL) in patients with PCOS, we may have identified a mitigable factor that could be increased with consumption of vitamin D-rich foods, exposure to sunlight, or with supplementation of vitamin D, thus leading to improved symptomatology associated with PCOS. Studies in adult women demonstrated positive effects of 25(OH)D supplementation on biochemical and clinical parameters of PCOS [29–31].

As expected, we found that in in the PCOS group various serum androgens were elevated, the prevalence of oligomenorrhea was increased, and ovarian appearance was with more peripherally distributed follicles compared to the non-PCOS subjects. Our adolescent Latina patients had lower AMH in the adjusted analysis, which was consistent with previous reports in adult women [50, 51], though prior studies have questioned the clinical significance of this in regard to increased risk of early menopause for Latinas [52, 53]. Additionally, 25(OH)D was lower in Latina adolescents with PCOS, albeit not reaching the null hypothesis rejection threshhold. Further research is needed to determine if establishing different AMH and 25(OH)D cutoffs for the diagnosis of PCOS in Latina adolescents would be required.

Our study limitations include its retrospective nature of data collection, a convenience sample of subjects from a single center, and incomplete data. For the latter, we compared subjects with and without missing observations, and did not observe systematic differences with respect to important demographic and clinical characteristics. While we did not specifically power our study to examine other risk factors for PCOS, the strength of this study is that it is the largest comparison of AMH and 25(OH)D in adolescent girls with PCOS to date. This study included a typical PCOS population and a comparator group composed of adolescent girls at risk for PCOS. Past studies have used control groups of healthy girls with regular menses and no hyperandrogenism [37, 54], or obese girls with regular menses and no hyperandrogenism [25], neither a good comparison to their PCOS counterparts due to potentially severe imbalance in confounders.

Future directions of work may include re-consideration of AMH’s use as a potential biomarker for PCOS in adolescents. Since there is such homogeneity in the findings of a discriminatory AMH level to identify PCOS in adolescents using various comparator groups, a meta-analysis of adolescent studies may identify a specific AMH cutoff. The most recent international guidelines for the adolescent population preclude use of ultrasound to diagnose PCOS for 8 years after menarche [5], however the diagnosis of PCOS and workup for the sequelae of the disease cannot wait 8 years – this may put these girls at increased risk of fertility issues, hypertension, diabetes, hyperlipidemia, and endometrial cancer [3, 4, 55]. Serum AMH would therefore be a non-invasive test with biologic plausibility to act a surrogate marker of ovarian follicle overabundance expressed in adolescents with PCOS. More research, such as an adequately powered randomized placebo-controlled clinical trial, needs to be done to properly evaluate the effect of 25(OH)D supplementation on mitigation and resolution of PCOS-associated signs, symptoms, and aberrant laboratory values in adolescents. In such a trial, AMH may play a unique role as a marker of PCOS as well as of the progress of treatment.

## Data Availability

The datasets used and/or analysed during the current study are available from the corresponding author on reasonable request.

## Abbreviations

25(OH)D: 25-hydroxyvitamin D
AGEC: Adolescent gynecology and endocrinology clinic
AMH: Anti-Müllerian hormone
AUC: Area under the curve
BMI: Body-mass index
CI: Confidence interval
DHEA-S: Dehydroepiandrosterone-sulfate
IRB: Institutional Review Board
PCOS: Polycystic ovarian syndrome
